# Based on small molecules: development and application of fibroblast activation protein inhibitors radiopharmaceutical in tumor precision therapy

**DOI:** 10.3389/fphar.2025.1593380

**Published:** 2025-05-14

**Authors:** Yihui Luo, Haitian Fu, Chunjing Yu

**Affiliations:** ^1^ Wuxi School of Medicine, Jiangnan University, Wuxi, China; ^2^ Department of Nuclear Medicine, Affiliated Hospital of Jiangnan University, Wuxi, China

**Keywords:** fibroblast activation protein, FAP, fibroblast activation protein inhibitor, FAPI, targeted radioligand therapy, radiopharmaceutical

## Abstract

The discovery of biomarkers for malignant tumors is driving the development of new radiopharmaceuticals in nuclear medicine. The development and optimization of novel radiopharmaceuticals to occupy an increasingly important role in tumor diagnosis and treatment. In recent years, fibroblast activation protein (FAP) has gained attention as a promising tumor target due to its widespread expression across various tumors. FAP inhibitor (FAPI) radiopharmaceuticals are considered to be the most promising to be developed for targeting FAP due to their rapid and specific tumor targeting. This review briefly outlines the developmental history of FAP-targeted small-molecule enzyme activity inhibitors, highlighting the effective role of targeting molecules, linkers, and certain functional groups in the delivery of radioisotopes to cancerous tissues. These development strategies will serve as a reference for the further development and application of relevant radiopharmaceuticals. This review also delineates the progress on clinical FAPI as a radioisotope delivery vehicle for the targeted radioligand therapy of tumors and introduces the latest combination therapy involving FAPI radiopharmaceutical for tumor treatment. The findings provide novel therapeutic insights into the targeted radioligand therapy of tumors.

## 1 Introduction

Cancer constitutes a major cause of death and poses a serious threat to health, making the exploration of tumorigenesis and development mechanisms a focal point in tumor research. Genetic mutations are essential for cancer occurrence, yet the tumor microenvironment (TME) appears to play a more complex and significant role in cancer progression (Arneth, 2019). Initially, pericarcinoma cells were viewed as bystanders in cancer development; however, increasing research on cancer mechanisms suggests that certain non-malignant cells in the TME significantly influence tumor progression (de Visser and Joyce, 2023). During tumor progression, normal fibroblasts (NFs) are induced to transform into cancer-associated fibroblasts (CAFs) and become major components of the TME, upregulating various cell surface markers, including α-smooth muscle actin (α-SMA), platelet-derived growth factor receptors-β (PDGF-β), and fibroblast activation protein (FAP) (Yamamoto et al., 2023). FAP has gained attention due to its high expression in 90% of epithelial tumors and low expression in normal tissues (Sahai et al., 2020).

In recent years, the development of novel radioligands based on new biomarkers and the success in translating them into clinical applications have significantly advanced the field of nuclear medicine (Czernin et al., 2019). The development of new biomarker-targeted radioligands aims to exploit the accumulation effect of target-rich proteins or molecules in diseases, and radioligands selectively deliver radionuclides to lesion sites, “illuminating” the lesion or destroying lesion cells. This innovative model of diagnosis and treatment fosters new perspectives on precise disease management (Langbein et al., 2019). FAP, as a pan-tumor target, is consistently expressed in mesenchymal cells, potentially addressing tumor heterogeneity caused by genetic mutations in cancer cells. Radionuclide therapy targeting FAP may alleviate the problem of therapeutic resistance. Therefore, the gradual emergence of radiopharmaceuticals targeting FAP (Chen and Song, 2019; Sanchez-Garrido et al., 2016). The advancement and application of novel radiopharmaceuticals targeting FAP, such as FAP inhibitor (FAPI) derivatives, have also become a critical research focus in nuclear medicine (Calais, 2020).

**FIGURE 1:**
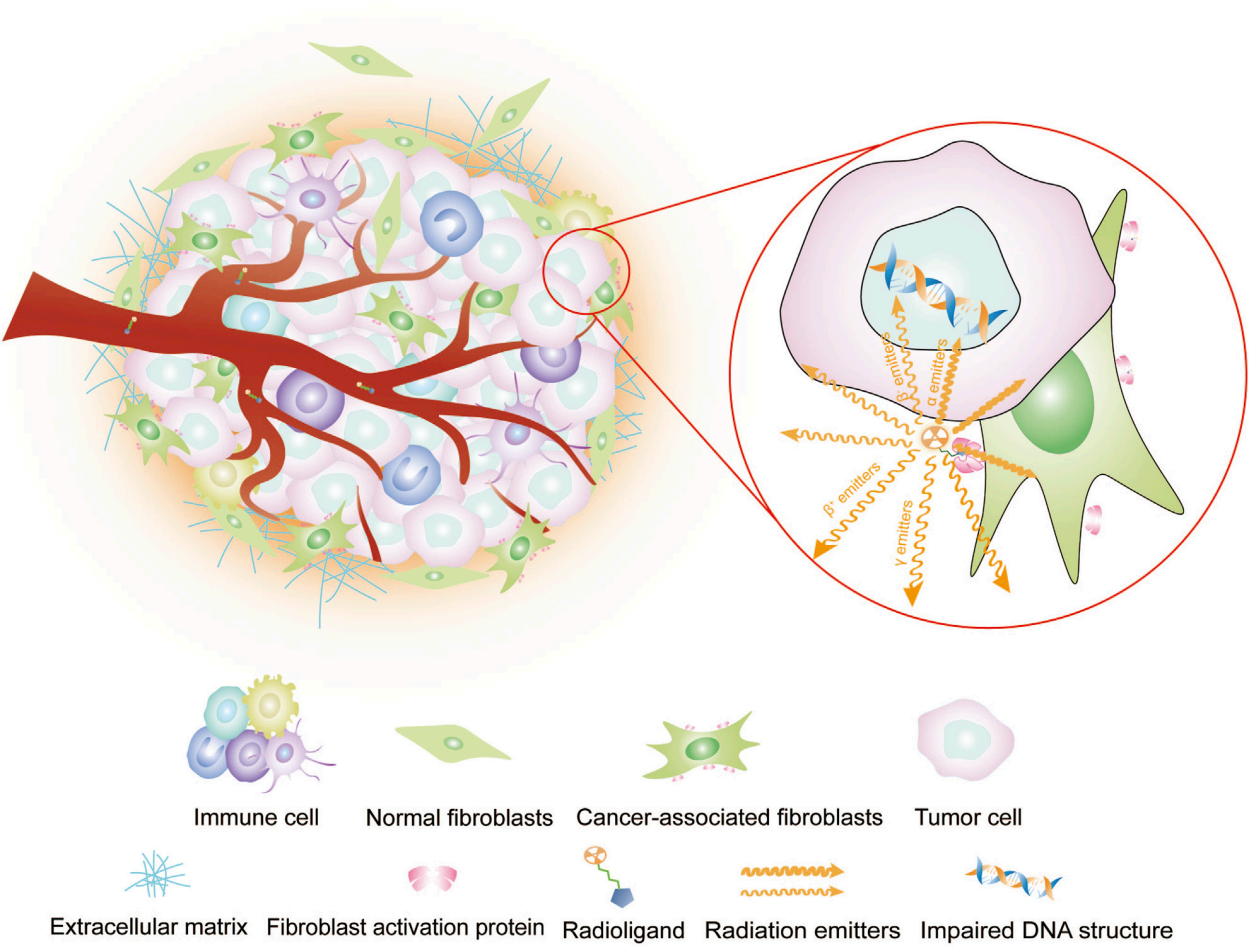
Imaging or intra-tumor RLT of radioligands targeting FAP. Radiopharmaceuticals carrying radioisotopes are typically administered intravenously via the bloodstream, so they reach their target location, i.e., the tumor. Therein, the radiopharmaceuticals and its target sites recognize each other and bind. Diagnostic radioisotopes with γ or β^+^ emitters, which are captured by computerized equipment for single-photon or positron imaging. Therapeutic radioisotopes are utilized to induce DNA damage for tumor cell destruction by emitting high-energy emitters, such as β^−^ emitters, which cause single-stranded DNA damage, or α emitters, which cause double-stranded DNA damage.

FAP-targeting radiopharmaceuticals do not directly target tumor cells but instead target CAFs, the core component of the tumor mesenchyme. Therefore, FAP-targeted imaging can accurately outline the tumor boundary of tumors with remarkably mesenchymal hyperplasia (Figure 1) (Kuten et al., 2021; Zhao et al., 2021). Increasing studies have demonstrated the effectiveness of FAPI as a PET tracer in diseases (Mori et al., 2023; Li M. et al., 2022), and some works indicate that FAPI PET/CT offers advantages over ^18^F-FDG PET/CT in solid tumor diagnosis, staging (Wegen et al., 2022; Zang et al., 2023; Promteangtrong et al., 2022; Pang et al., 2021). The publication of procedural standards and practice guidelines for FAPI PET signifies that FAPI PET has attained authoritative recognition, and the clinical application of FAPI PET molecular imaging is becoming standardized (Hope et al., 2024).

Targeted radioligand therapy (RLT) has gained recognition and demonstrated significant potential in clinical practice. Notable examples include ^177^Lu-PSMA for prostate cancer, ^90^Y-Pentixather for multiple myeloma, and ^177^Lu-DOTATATE for neuroendocrine tumors. This treatment employs radioisotopes combined with specific ligands to precisely target cancer cells. It has the potential to reduce the radiation dose to patients and minimize exposure to normal tissue compared with conventional radiotherapy. In targeting FAP for RLT, tumor cells are influenced through “cross effects” (Figure 1). Compared with other tumor biomarker-targeted radiopharmaceuticals, the pan-tumoral expression of FAP makes FAPI applicable to the RLT of a wide range of tumors.

In the development of FAPI-relevant therapeutic radiopharmaceuticals, the issues of intra-tumor retention, tumor targeting, biodistribution, and clinical translation still needs to be addressed. Targeted therapy with several FAPI radiopharmaceuticals has demonstrated encouraging outcomes in pain management for patients with advanced-stage tumors leading to enhancements in their quality of life. With the progress of precision medicine technology and the improvement of therapeutic radioisotopes industry chain, FAPI is expected to become a new means of tumor treatment. This review briefly outlines the developmental history of FAP-targeted small-molecule enzyme activity inhibitors, highlighting the effective role of targeting molecules, linkers, and certain functional groups in the delivery of radioisotopes to cancerous tissues. These development strategies will serve as a reference for the further development and application of relevant radiopharmaceuticals. This review also delineates the progress on clinical FAPI as a radioisotope delivery vehicle for the targeted radioligand therapy of tumors and introduces the latest combination therapy involving FAPI radiopharmaceutical for tumor treatment. The findings provide novel therapeutic insights into the targeted radioligand therapy of tumors.

## 2 FAP exerts pro-tumorigenic effects

FAP is a type II transmembrane protein and serves as a marker for classifying functional subtypes of CAFs. Some studies indicate that this protein is also expressed on the surface of certain tumor cells and macrophages (Mentlein et al., 2011; Wei et al., 2022). The high expression of FAP in the tumor mesenchyme influences the biological behavior of tumor cells and adjacent mesenchymal cells.

### 2.1 Effect of FAP on malignant cell

FAP has been demonstrated to promote malignant cell proliferation and invasion. Co-culturing studies with FAP+ cells demonstrate that the cancer cells exhibit increased proliferation and migration (Teichgraber et al., 2015; Higashino et al., 2019; Cai et al., 2023). Overexpression of FAP in cancer cells inhibits apoptosis and promotes proliferation and invasion of cancer cells, whereas downregulation of FAP expression decreases proliferation and invasion, promoting apoptosis (An et al., 2022).

### 2.2 Effects of FAP on TME

FAP promotes tumor angiogenesis by promoting neovascular sprouting and vascular co-option (Koczorowska et al., 2015; Qi et al., 2022). Additionally, FAP reduces extracellular matrix degradation (Bukhari et al., 2023), which may impede immune cell infiltration. FAP expression modulates immune cell status by inducing macrophage M2 polarization (Higashino et al., 2019; Cai et al., 2023). Depletion of FAP+ CAFs enhances T cell infiltration in the tumor (Xiao et al., 2023). Treatment with CAR-T cells targeting FAP remodels the TME and increases CD8^+^ T cells survival (Liu et al., 2023). In conclusion, FAP expression in the mesenchyme reduces tumor killing by immune cells, and the depletion of FAP in combination with anti-tumor therapy will improve therapeutic efficacy.

### 2.3 Prognostic impact of high FAP expression in clinical patients

In several clinical analyses, FAP+ CAFs are associated with tumor metastasis and poor patient survival (Zhao Y. et al., 2024). FAP contributes to resistance to neoadjuvant chemotherapy and poor prognosis by inducing mesenchymal transformation and cancer stem cell transformation (Zhao and Zhu, 2023).

The biological function of FAP in tumors remains incompletely understood, although most studies indicate that it plays a role in tumor progression through multiple mechanisms.

## 3 History of specific FAP inhibitor development

FAP belongs to the serine protease family, and shares 51% amino acid sequence similarity with dipeptidyl peptidase IV (DPP IV). It exhibits similar endopeptidase functions to prolyl endopeptidase (PREP) (Park et al., 1999). A significant challenge in developing FAP-targeting inhibitors is achieving selectivity for the DPPs and PREP. Although enzyme activity inhibitors based on boric acid have demonstrated effective inhibition of FAP in preclinical studies (Tran et al., 2007), neither the Phase II trial nor the combination therapy with talabostat showed beneficial clinical effects (Eager et al., 2009a; Eager et al., 2009b; Cheng et al., 2005; Engel et al., 2006).

**FIGURE 2:**
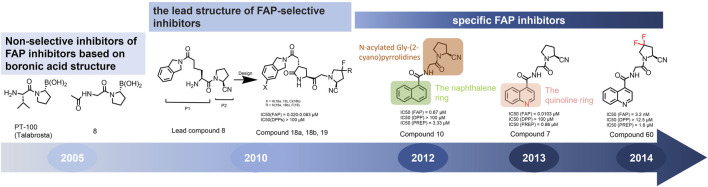
Timeline of FAP inhibitors development. In 2005, Tran et al. (2007) evaluated the inhibitory effect of PT100 (talabostat) on the enzymatic activities of FAP and DPPs and compound 8 on the enzymatic activities of FAP and PREP by analyzing the substrates of DPP IV, PREP, and FAP. In 2010, Tsai et al. (2010) determined the effectiveness of pyrrolidine moieties containing a carbonitrile structure as FAP inhibitors by optimizing lead compound 8, a non-selective DPP IV inhibitor. In 2012, Ryabtsova et al. (2012) identified N-acylated Gly-(2-cyano) pyrrolidines with significant potential for FAP specific recognition. In 2013, Jansen et al. (2013) discovered of a new class of FAP inhibitors with a N-(4-quinolinoyl)-Gly-(2-cyanopyrrolidine) scaffold and improved FAP selectivity. In 2014, Koen Jansen’s team has enhanced the fluorination of core structural pyrrolidines, thereby improving their FAP selectivity.

In 2010, Ting-Yueh Tsai’s team developed a series of compounds optimized with non-selective DPPIV inhibitors and screened them for targeting FAP, they identified dipeptide-derived carbonitrile as a selective inhibitor of FAP (Figure 2) (Tsai et al., 2010). In 2012, by selecting the carbonitriles scaffold, Ryabtsovas’ team investigated the effects of N-acyl group and structural modification of the 2-cyanopyrrolidine residue on FAP selectivity by selecting the carbonitrile scaffold, Compound 10 contains a naphthalene ring scaffold connecting to the N-acylated Gly-(2-cyano) pyrrolidine structure (Figure 2), which exhibited high selectivity for FAP and low selectivity for other DPPs, serving as the foundational structure for subsequent FAP inhibitors development (Ryabtsova et al., 2012). In 2013, Jansen’s team optimized the structure of Compound 10 by substituting nitrogen at different sites in the naphthalene ring to influence electron cloud density in the aromatic ring and regulate ligand to target binding. These product with increasing affinity for FAP from micromolar to nanomolar levels. Among them, Compound 7 (Figure 2) features an N-(4-quinolinyl)-glycyl-(2-cyanopyrrolidine) scaffold, demonstrated reduced selectivity for DPPs and exhibited an 83-fold higher affinity for FAP than for PREP, making it a promising candidate for targeting FAP inhibitors (Jansen et al., 2013). In 2014, their team further optimized the structure of Compound 7 and revealed that substituting other amino acids for glycine residues significantly diminished the FAP potency of each substituted residue. They discovered Compound 60 with pyrrolidinyl fluorination (Figure 2), which showed a 562-fold higher affinity for FAP than for PREP whereas maintaining low selectivity for the DPPs (Jansen et al., 2014).

## 4 FAPI emerges as radioligands in nuclear medicine (from FAPI-01 to FAPI-46)

**FIGURE 3:**
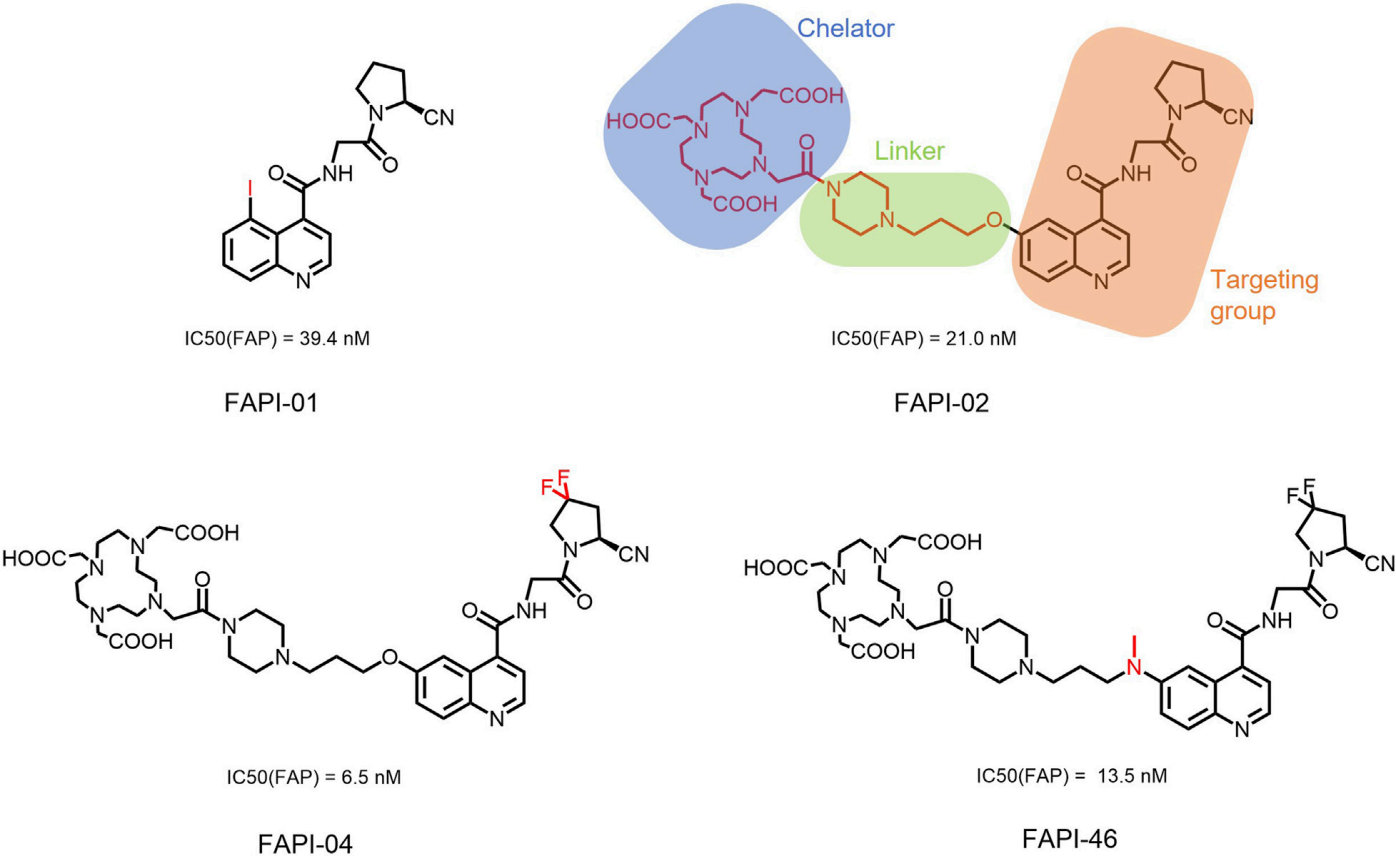
Chemical Structure of FAPI-01, FAPI-02, FAPI-04, and FAPI-46. I^125^ was labelled by iodination to constitute the FAPI-01 structure for visualization of the binding effect to FAP *in vitro* and distribution *in vivo*. However, enzymatic deiodination led to radionuclide off-targeting. The chelator DOTA of chelating radionuclide groups was introduced into the FAP targeting group by using a piperazine ring and a hydrocarbon chain as the connecting arm to form FAPI-02. The ring chelator can form a solid complex with radionuclides, such as ^68^Ga and ^177^Lu, therefore, ^68^Ga-FAPI-02 has a favorable and stable tumor imaging effect. The design of FAPI-04 was optimized though the pyrrolidinyl fluorination of the targeting group. The hydrocarbon chain of the linker was structural modified to form FAPI-46 with enhanced tumor uptake and biodistribution.

In 2018, Loktev’s team developed targeted FAP tracers by performing radionuclide labeling on the structure of Compound 7 naming them FAPI-01 and FAPI-02 (Figure 3). FAPI-01 was directly labeled with I^125^, FAPI-02 was labeled with gallium-68 using the chelator DOTA (1,4,7,10-tetraazacyclododecane-1,4,7,10-tetraacetic acid), which can form stable complexes with radioisotopes such as ^68^Ga^3+^ or ^177^Lu^3+^. This change resulted in little change in ligand binding affinity for the target protein, which remained at the nanomolar level. Whereas FAPI-02 exhibited superior binding ability *in vitro* cell experiment. Imaging with ^68^Ga-FAPI-02 in tumor patients revealed high uptake of tumor foci within 10 min of administration and low background activity, particularly in the brain and liver organs. The uptake in tumor lesions exhibited a maximum at 1 h, followed by a decline over the subsequent 3 h. The time-dependent reduction of radioactivity in tumors limits the use of FAPI-02 in tumor therapy (Loktev et al., 2018).

To enhance tumor uptake and retention time, the team optimized the design based on the structure of FAPI-02, adjusting the pharmacokinetics of the radiopharmaceuticals through hydrogen and fluorine substitution on the pyrrole, while analyzing the conformational association between the chelating and the targeting groups. They screened FAPI-04 with higher affinity to FAP, which demonstrated optimal tumor and non-target tissue distribution in preclinical imaging experiments, showing a longer retention time in tumor compared to FAPI-02. Imaging with ^68^Ga-FAPI-04 in the patients with tumors maintained high image contrast at 3 h post-injection (Lindner et al., 2018). As clinical use of ^68^Ga-FAPI-04 increases, its effectiveness as a tracer in PET imaging is gradually being recognized. Concurrently, the enhancement of tumor uptake and intra-tumor retention time through chemical structure modification of FAPI for oncology theranostics remains an active area of research in radiopharmaceutical development.

Chemical modifications from FAPI-02 to FAPI-04 prolonged tumor retention, reducing the 75% elution rate from 1 to 3 h of tumor uptake to 50%. To deliver higher doses to tumors and improve the therapeutic utility of FAPI. Anastasia Loktev’s team developed FAP targeting molecules by further optimizing FAPI-04 to increase radio-dose in tumor whereas maintaining low non-specific binding to normal tissues. They modified the piperazine ring and substituted elements in the hydrocarbon chain in the structure of FAPI-04, screening FAPI-46. Although FAPI-46 had a slightly lower affinity for FAP binding than FAPI-04, ^68^Ga-FAPI-46 showed lower uptake in all organs than ^68^Ga-FAPI-04 and increased tumor uptake (Loktev et al., 2019). This compound is considered to have greater therapeutic potential in tumor therapy. Subsequent evaluations of ^225^Ac/^177^Lu-FAPI-46 in tumor models showed limited therapeutic efficacy. FAPI-04 and FAPI-46 established a foundation for the development of later-stage FAPI derivatives (Liu et al., 2022).

## 5 Development of FAPI therapeutic radiopharmaceuticals

FAPI derivatives are a class of small molecules that exhibit high selectivity for FAP. The high affinity and selectivity of the targeting group for FAP confer FAPIs with the ability to facilitate rapid distribution and targeted tumors. FAPIs have a smaller molecular weight and are less likely to be antigenic than antibodies. This makes them suitable to carry radioisotopes to tumors for RLT. However, the rapid *in vivo* clearance of FAPI also leads to insufficient tumor uptake, thereby rendering the limited efficacy of RLT (van den Hoven et al., 2023). The application of targeting FAP for RLT has made rapid strides due to the optimization of targeting group selectivity, linker technology, the side chains strategy, and multivalent strategy (Galbiati et al., 2024; Zhang et al., 2021; Cui et al., 2024; Yu et al., 2024).

**FIGURE 4:**
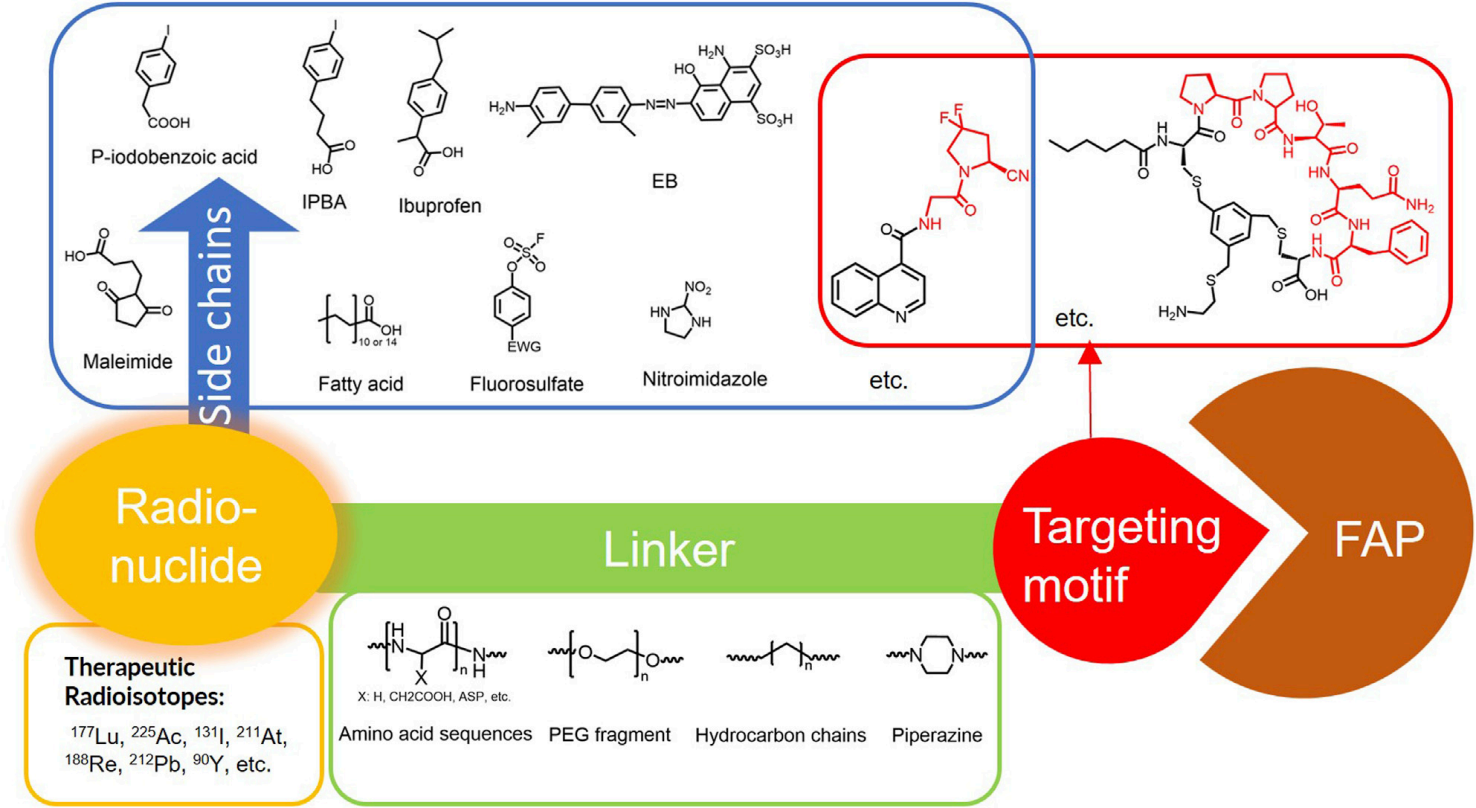
Design of radiopharmaceutical chemistry targeting FAP. The three essential elements to the design of an effective FAP inhibitor radiopharmaceutical are as follows: (1) An effective targeting motif that requires favorable FAP binding and high FAP selectivity. (2) A stable linker to prevent radionuclide off-targeting *in vivo*. PEG fragments, hydrocarbon chains and amino acid sequences are commonly used as linker in the development of FAPIs. (3) Radioisotopes are selected according to clinical needs, such as ^18^F, ^68^Ga and ^89^Zr for PET imaging, ^99m^Tc and ^111^In for SPECT imaging, ^177^Lu and ^225^Ac for RLT. Introducing chelating groups is the main strategy in the design and development of FAPI to combine radionuclides. Side chain groups mainly regulate the pharmacokinetics of the drug and increase the binding effect of the ligand to the target in order to optimize the distribution of the radiopharmaceutical in the body (red areas of targeting motifs indicate FAP-specific recognition regions).

The effective development of FAPI derivatives is contingent upon the meticulous determination of targeted molecules, linkers, and chelators (Figure 4). The targeting molecule must maintain effective binding with its target, the linker must be sufficiently stable, and the chelator must form a solid complex with the radioisotope. Cyclic chelators exhibit superior binding efficacy to radiometals, forming more stable complexes compared with acyclic complexes, so DOTA and DOTAGA were frequently used in radiopharmaceutical development (Rong et al., 2023; Price and Orvig, 2014).

### 5.1 Modification of targeting motif

Cyclic peptides exhibit higher stability and biological effects than linear peptides. Therefore, the design of cyclic peptide structures as new FAP targeting motifs may address the deficiencies of FAPI. Zboralski’s team designed a cyclic peptide known as FAP-2286, which possesses an FAP-specific binding region of the sequence Pro-Pro-Thr-Gln-Phe. Two cysteines (Cys) are used as linkers to form a cyclic bridge to enhance the stability of the molecule. Other structures been added to the molecule to enhance targeting to FAP through the spatial conformation. The results showed that FAP-2286 (IC_50_ = 2.7 nM) demonstrated similar potency to FAPI-46 (IC_50_ = 13.5 nM) in inhibiting the enzymatic activity of FAP and favorable selectivity for DPP4 (IC_50_ > 10,000 nM) and PREP (IC_50_ > 1,000 nM). ^177^Lu-FAP-2286 exhibited improved tumor uptake and biodistribution in HEK-FAP tumor-bearing mice, significantly prolonging the retention time in cancer compared to ^177^Lu-FAPI-46. The survival time of tumor-bearing mice treated with 60 MBq of ^177^Lu-FAP-2286 was significantly extended (Zboralski et al., 2022). Although FAP-2286 showed promising anti-tumor activity in tumor models, significant renal uptake was observed in patient imaging evaluations of ^68^Ga-FAP-2286 (Pang et al., 2023a). Thus, an assessment of possible nephrotoxicity from its RLT is necessary.

### 5.2 Chemical modification of linkers

The linker, a critical structural component that connects the chelating group to the targeting moiety, must exhibit sufficient *in vivo* stability to prevent premature radionuclide dissociation and subsequent off-target radiation exposure in healthy tissues. By modulating the length, composition, and rigidity of the linker, researchers can optimize pharmacokinetic properties, including circulation time and biodistribution, while maintaining the ligand’s binding affinity for its target. In the development of FAPIs, commonly employed linkers include: polyethylene glycol (PEG) chains, amino acid or short peptide spacers, hydrocarbon-based, and so on.

Du’s team modified OncoFAP, which was developed by Backhaus’ team (Backhaus et al., 2021), designing DOTAGA-FAPI-FUSCC-I and DOTAGA-FAPI-FUSCC-II using glycine repeat sequence as the linker (Du et al., 2024). The affinity was slightly improved compared to FAPI-04. Preclinical results indicated that DOTAGA-FAPI-FUSCC-II with glycine tripeptide had higher tumor uptake than DOTAGA-FAPI-FUSCC-I with glycine dipeptide. The uptake of ^177^Lu-DOTAGA-FAPI-FUSCC-I and ^177^Lu-DOTAGA-FAPI-FUSCC-II showed a significant trend of decreasing radioactivity in HT-1080-FAP tumors at 24 h, and both radiopharmaceuticals exhibited similar tumor uptake from 1 h to 24 h, with an in-tumor retention of approximately 20% at 24 h. Therapeutic efficacy was not further evaluated.

Through docking simulation studies, Huang’s research team designed and evaluated the binding affinity of four FAP-targeting ligands (DOTA-FAPI-04, DOTA-PEG_2_-FAPI, DOTA-GlcP-FAPI, and DOTA-FAPT) to FAP (Huang et al., 2024). The results indicated that DOTA-FAPT had the lowest binding energy (−9.2 kcal/mol), suggesting it had the strongest affinity for FAP. *In vivo* experiments demonstrated that the uptake and retention of ^177^Lu-FAPT in tumors were significantly higher than those of ^177^Lu-FAPI-04. The tumor areas under the curve (AUC) value of ^177^Lu-FAPT was twice that of ^177^Lu-FAPI-04, implying that ^177^Lu-FAPT accumulates more in tumors, which may enhance the therapeutic effect. ^177^Lu-FAPT exhibited significant efficacy in inhibiting tumor growth, particularly the 37 MBq dose group, which showed the best tumor growth inhibition and survival. This study indicates that ^177^Lu-FAPT is a promising therapeutic for tumors in the future.

The modification of the linker is often accompanied by a modification of the side chain, so we discuss this in more detail later on.

### 5.3 Chemical modification by adding side chains

FAPI exhibits rapid clearance *in vivo*, resulting in a low in-tumor radiation dose and limiting its applications in oncology therapy. Alterations in pharmacokinetics through side chains enhance ligand-target binding. The albumin-binding motif (ABM) strategy leverages reversible interactions between small molecules and plasma albumin, thereby reducing renal clearance and prolonging circulatory half-life. This approach enhances tumor accumulation by promoting passive targeting through the enhanced permeability and retention effect. Notably, the ABM strategy has applications in the development of other radiopharmaceuticals to improve radioligand therapy outcomes (Lau et al., 2019). Commonly employed ABM modifications include: the p-iodophenyl moiety (IPBA), fatty acids, and truncated Evans blue (EB), among others. The remarkable potential of covalent modification strategies in radiopharmaceutical development. This approach involves the rational design of ligands capable of forming irreversible covalent bonds with nucleophilic residues in secondary binding pockets of target proteins, resulting in a stronger binding effect of the ligand to the target protein and also improving the retention of the ligand in the tumor.

#### 5.3.1 The p-iodophenyl moiety (IPBA)

Lindeman’s team selected the affinity portion of a fluorescent FAP-targeted small molecule tracer (Mukkamala et al., 2022) developed in prior research as the FAP target moiety to linked with the chelator DOTA, naming FAP6-DOTA, which incorporates a PEG_5_ fragment and a 1,2,3-triazole ring as the linker. However, the SPECT in tumor-bearing mice revealed a highly radioactive background, and biodistribution studies indicated elevated radioactivity in the liver and spleen. The team then designed FAP6-IP-DOTA by introducing IPBA, significantly reducing non-specific uptake in the liver, spleen, and kidney, whereas increasing the retention time of the radiopharmaceuticals in circulation and enhancing tumor uptake. Different radionuclide-labeled forms of the same molecule exhibited varying intra-tumor retention effects. ^111^In-FAP6-IP-DOTA demonstrated approximately 55% intra-tumor retention at 24 h. In contrast, ^177^Lu-FAP6-IP-DOTA peaked at 1 h in 4T1 tumors, with only 18% tumor retention at 24 h. Injection of ^177^Lu-FAP6-IP-DOTA showed tumor growth slowed in several models (Lindeman et al., 2023). Subsequently, the team continued to optimize the design of the *ex vivo* biodistribution of 7 compounds utilizing glycine, PEG fragments, hydrocarbon chains, piperazine, 1,2,3-triazole, p-hydroxybenzoic acid as linkers, and 4-para fluorine, methyl substitution of IPBA in the structure (Lindeman et al., 2024). The results indicated that FAP6-19, with IPBA and piperazine as linkers, exhibited an upward trend in intra-tumor uptake at 24 h, with the intra-tumor uptake at 120 h being 57% of that at 24 h (Table 1), and significantly prolonged intra-tumor retention time. However, anti-tumor efficacy was not further investigated in the study.

**TABLE 1:** Optimization strategy and evaluation effect of radiopharmaceuticals.

The optimal molecule in the study	Development strategies	Chemical structure	Binding affinity	Animal models	The retention of tumor (% ID/g)	Ref.
FAP-2286	Targeting motif (cyclic peptide)	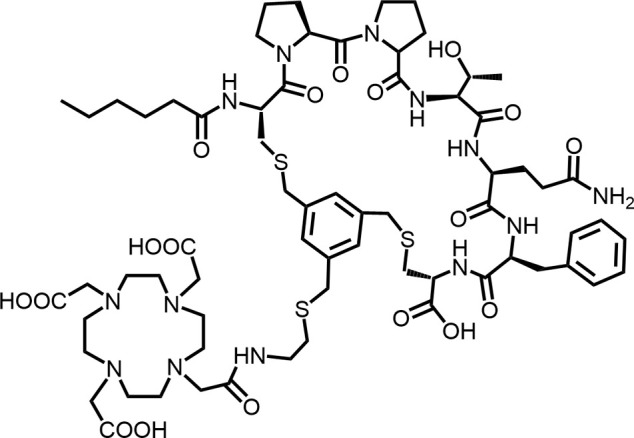	IC50 = 2.7 nM KD = 1.1 nM	HEK-FAP	^177^Lu-FAP-2286 3 h p.i. 21.1 ± 4.4 72 h p.i.16.4 ± 3.6	Zboralski et al. (2022)
DOTAGA-FAPI-FUSCC-II	Linker (glycine repeat sequence)	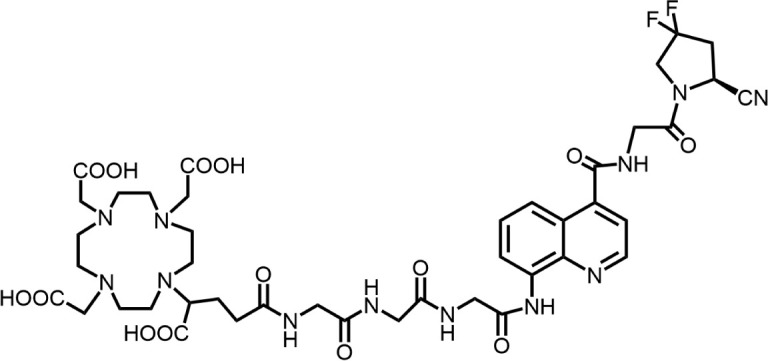	IC50 = 0.13 nM	HT-1080-FAP	^177^Lu-FAPI-FUSCC-II 1 h p.i. 7.2 ± 0.68 24 h p.i. 1.37 ± 0.17	Du et al. (2024)
FAPT	Linker (GlcP-PEG2)	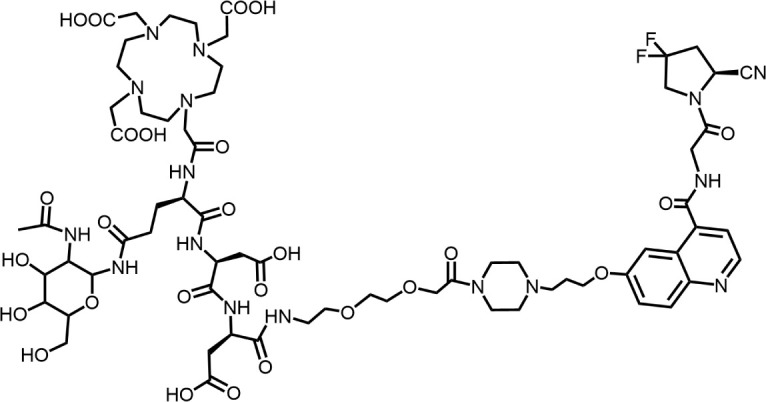	IC50 = 0.42 nM	A549-FAP	^177^Lu-FAPT 4 h p.i. 26.68 ± 6.8 gradually decreased after 24 h	Huang et al. (2024)
FAP6-IP-DOTA	Targeting motif (FAP6) Linker (PEG fragment and 1,2,3-triazole ring) ABM (IPBA)	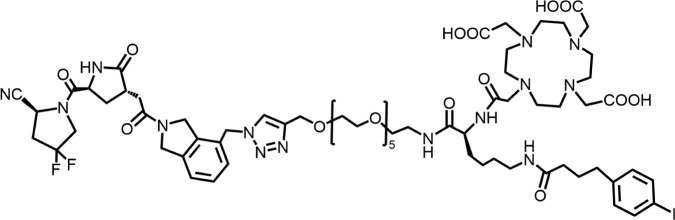	Kd = 0.92 nM	4T1	^177^Lu-FAP6-IP-DOTA 1 h p.i. 12.15 ± 1.61 24 h p.i. 2.16 ± 0.29 120 h p.i. 0.54 ± 0.04	Lindeman et al. (2023)
FAP6-19	Targeting motif (FAP6) Linker (piperazine and benzene) ABM (IPBA)	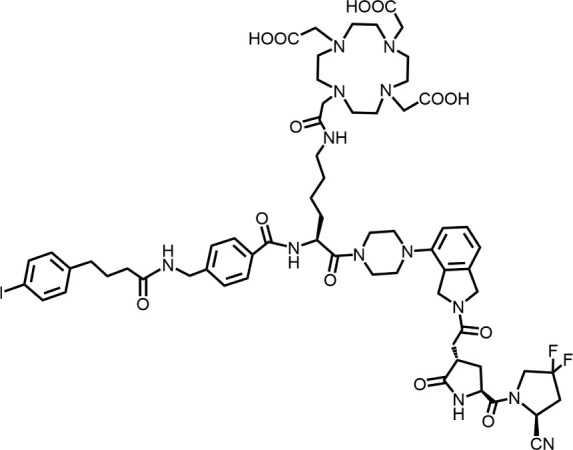	Kd = 18.2 nM	4T1	^177^Lu-FAP6-19 3 h p.i.4.29 ± 0.72 24 h p.i.5.96 ± 0.42 120 h p.i.3.42 ± 0.59	Lindeman et al. (2024)
TE-FAPI-04	ABM (IPBA)	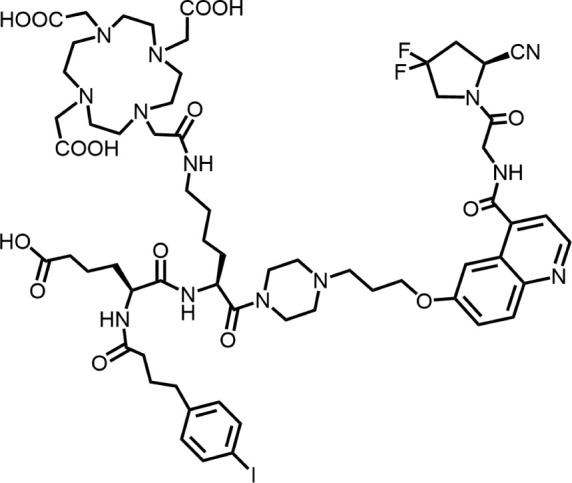	IC_50_ = 9.43 nM	HT-1080-FAP	^177^Lu-TE-FAPI-04 24 h p.i. 3.86 ± 1.15	Ding et al. (2022)
TEFAPI-06	ABM (IPBA)	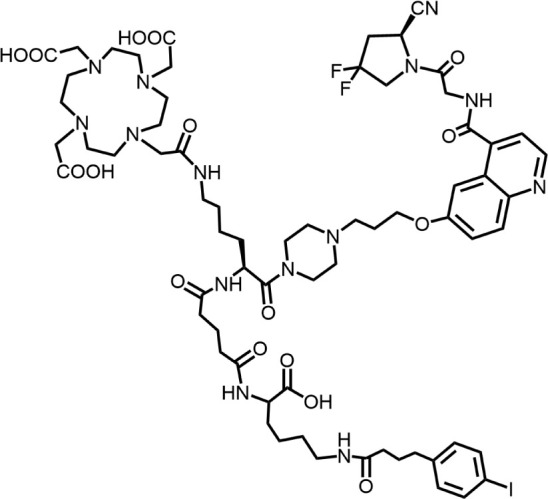	IC_50_ = 12.24 nM Kd = 10.16 nM	Pancreatic cancer PDX	^177^Lu-TEFAPI-06 24 h p.i. 8.68 ± 0.73 96 h p.i. 7.33 ± 2.28	Xu et al. (2022)
FSDD_0_I	Linker (peptide sequence) ABM (IPBA)	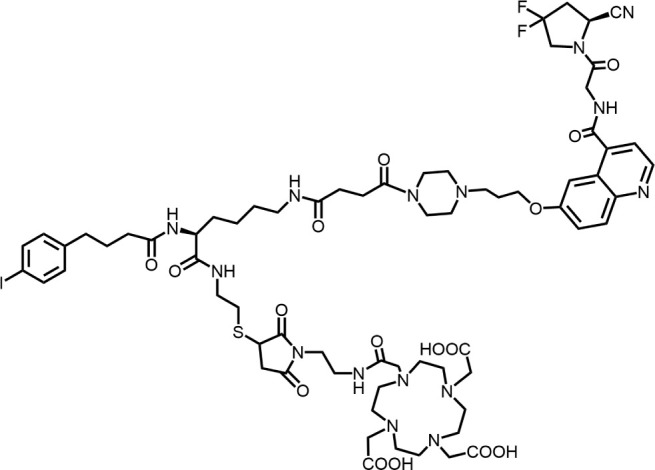	IC_50_ = 14.21 nM	HCC-PDX	^177^Lu-FSDD_0_I 4 h p.i. 18.50 ± 0.50 24 h p.i. 7.83 ± 1.19	Meng et al. (2022)
RPS-309	Linker (PEG fragment, amino acid) ABM (p-iodobenzoic acid)	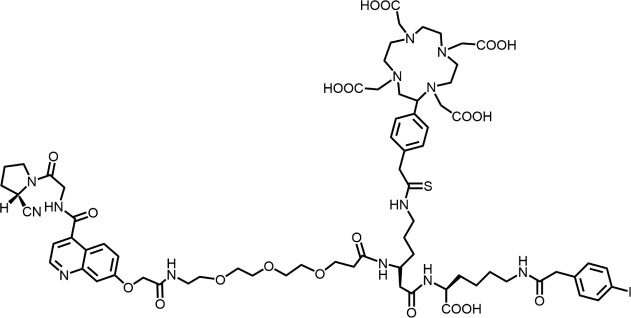	IC_50_ = 7.30 nM	SW872	^177^Lu-RPS-309 4 h p.i. 4.8 ± 0.6 24 h p.i. 1.7 ± 0.2 96 h p.i. 0.3 ± 0.03	Kelly et al. (2021)
EB-FAPI-B1	Linker (PEG fragment) ABM (EB)	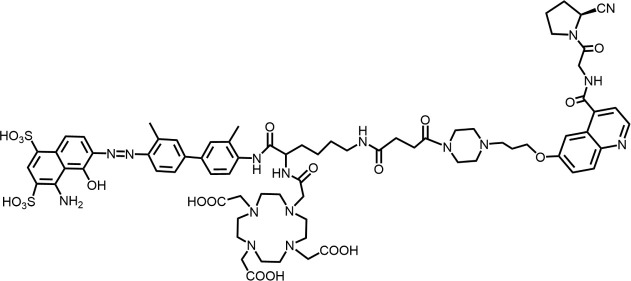	IC_50_ = 16.50 nM	U87	^177^Lu-EB-FAPI-B1 96 h p.i. 12.42 ± 1.54	Wen et al. (2022)
FAPI-46-EB	ABM (EB)	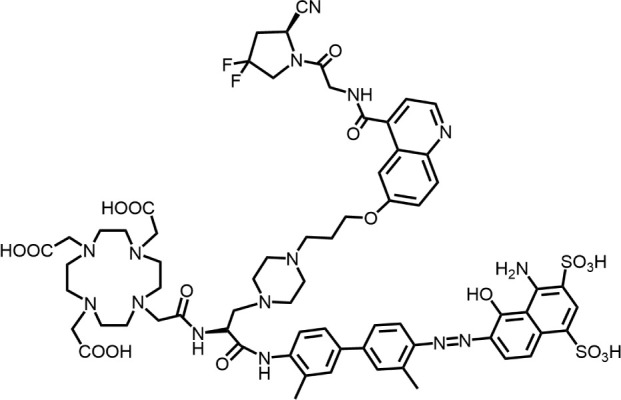	IC_50_ = 634.3 pM	HEK293.hFAP	^177^Lu-FAPI-46-EB 4 h p.i. 12.58 ± 2.83 24 h p.i. 16.69 ± 1.01 168 h p.i. 6.75 ± 1.06	Millul et al. (2023)
FAPI-C16	ABM (palmitic acid)	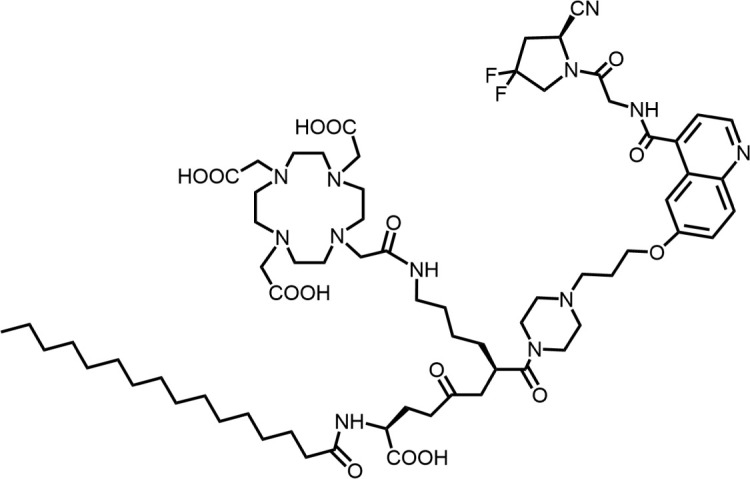	IC_50_ = 5.06 nM	HT-1080-FAP	^177^Lu-FAPI-C16 24 h p.i. 11.23 ± 1.18 72 h p.i. 6.50 ± 1.19	Zhang et al. (2021)
DOTA-FAPI-maleimide	Linker (PEG fragment) ABM (maleimide)	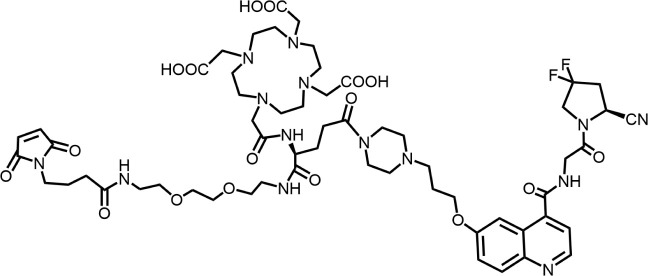	IC_50_ = 1.20 nM Kd = 0.70 nM	HT-1080-FAP	^177^Lu-DOTA-FAPI-maleimide 24 h p.i. 5.04 ± 1.67 4 days p.i. 3.40 ± 1.95	Feng et al. (2024)
FAPI-Ibu1	Linker (amino acid) ABM (ibuprofen)	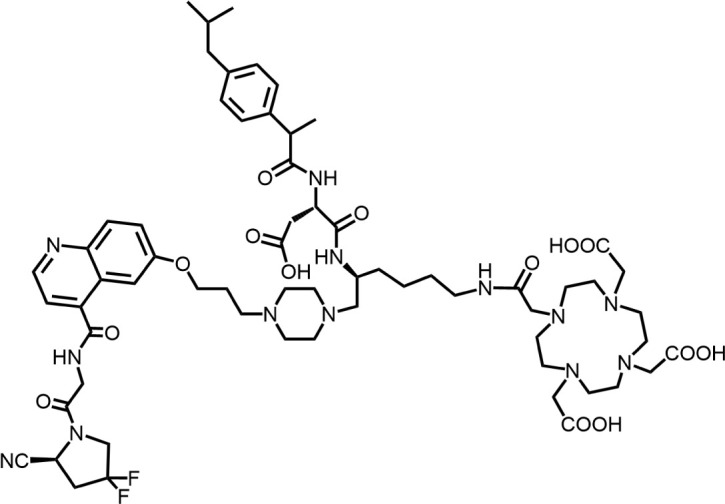	IC_50_ = 1.17 nM	U87MG	^177^Lu-FAPI-Ibu1 4 h p.i. 14.21 ± 5.31 24 h p.i. 4.75 ± 0.46 168 h p.i. 0.40 ± 0.37	Zhou et al. (2025)
FAPI-mFs	Covalent strategy (sulfone fluoride)	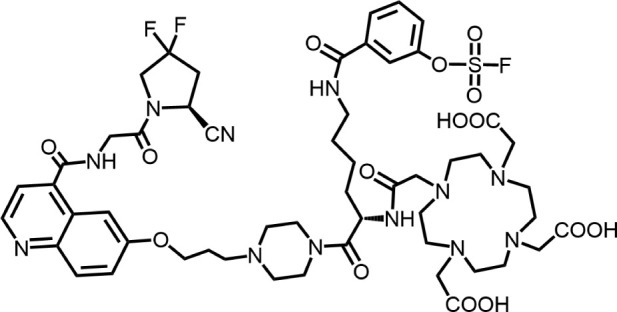	IC_50_ = 1.4 nM	HT-1080-FAP	^177^Lu-FAPI-mFs No specific figures in article	Cui et al. (2024)
1	Nitroimidazole	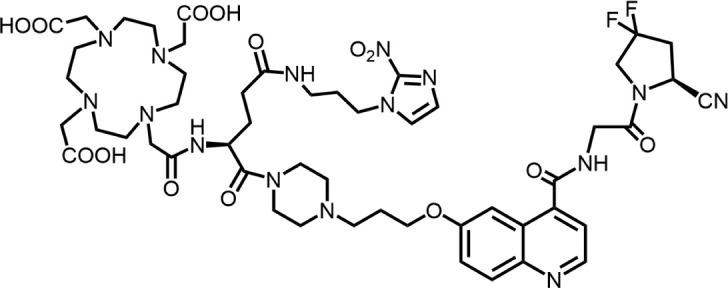	IC_50_ = 7.44 nM	U87MG	^177^Lu-1 1 h p.i. 41.09 ± 5.46 2 h p.i. 50.75 ± 3.87	Luo et al. (2024)
DOTA-PEG-PDA-FAPI	Polydopamine nanocarriers	Nano-materials	—	U87MG	^177^Lu-DOTA-PEG-PDA-FAPI Intra-tumoral injection day 5: 182.6 ± 73.8	Wang et al. (2025)
BiOncoFAP	Dimerization	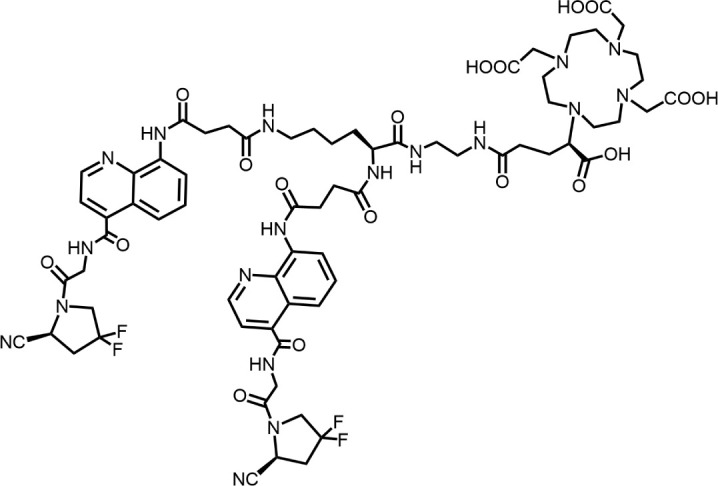	IC_50_ = 0.17 nM KD = 0.78 nM	HT-1080.hFAP	^177^Lu-BiOncoFAP 4 h p.i. 31.18 ± 3.7 24 h p.i. 19.21 ± 5.42 48 h p.i. 16.46 ± 3.18	Galbiati et al. (2022)
TriOncoFAP-DOTAGA	Multimerization	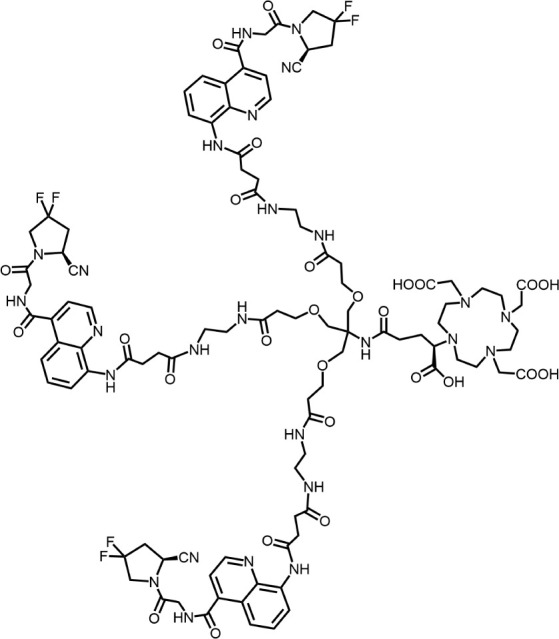	IC_50_ = 13 pM	SK-RC-52.hFAP	^177^Lu-TriOncoFAP-DOTAGA 24 h 43 96 h 18	Galbiati et al. (2024)
DOTA-Suc-Lys-(FAPI-04)_2_	Dimerization	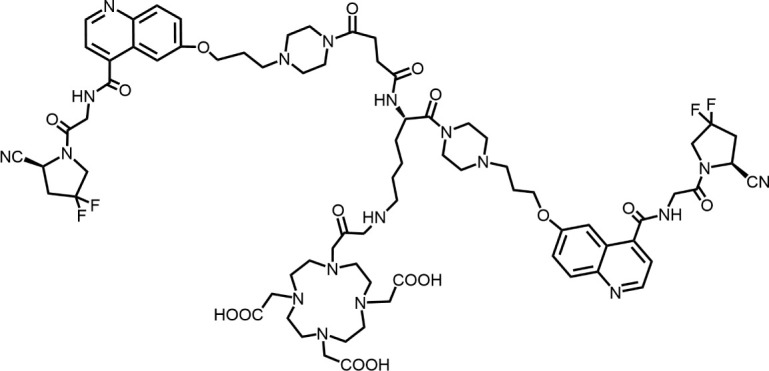	—	SKVO3	^177^Lu-DOTA-Suc-Lys-(FAPI-04)_2_ 1 h p.i. 6.63 ± 0.57 24 h p.i. 1.47 ± 0.15	Zhong et al. (2023)
ND-bisFAPI	Dimerization	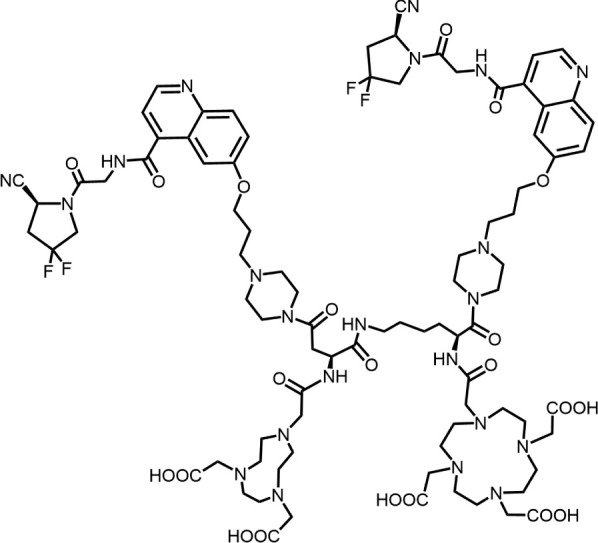	IC_50_ = 2 nM	A549-FAP	^177^Lu-ND-bisFAPI 4 h p.i. 12.3 ± 4.2 24 h p.i. 5.0 ± 0.6 168 h p.i. 0.3 ± 0.2	Li H. et al. (2022)
DOTA-4P(FAPI)_4_	Multimerization	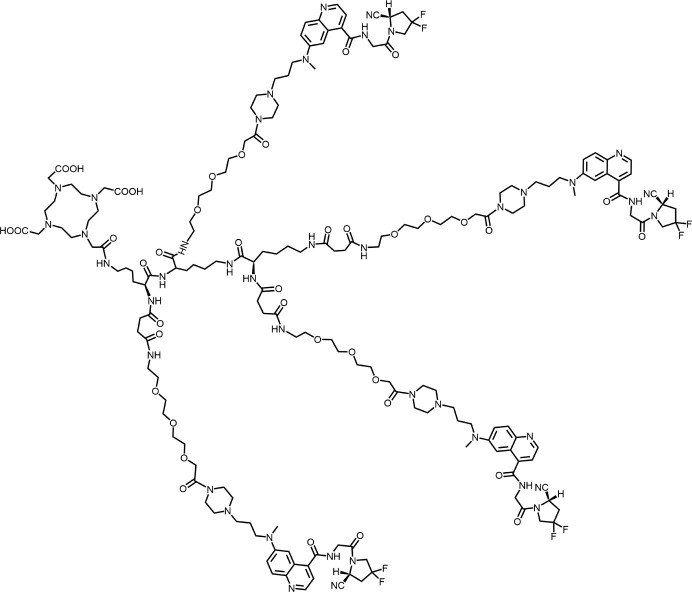	IC_50_ = 15.56 nM	HT-1080-FAP	^177^Lu-DOTA-4P(FAPI)4 24 h p.i. 21.4 ± 1.7 96 h p.i. 14.8 ± 0.9	Pang et al. (2023b)

The four molecules TE-FAPI01-04, designed and developed by Liu’s team through the introduction of IPBA, and varying short amino acid chains as linkers, exhibit a decreasing trend in radioactivity within tumors over 12 h. Among these structures, ^177^Lu-TE-FAPI-04 demonstrates the highest tumor uptake and shows the most promise for further development (Ding et al., 2022). The team subsequently designed TEFAPI-06 and TEFAPI-07 by comparing the introduction of both IPBA and truncated EB regarding the overall effect of the radiopharmaceuticals (Xu et al., 2022). The two compounds possess similar affinity, and the patient-derived xenograft (PDX) model of pancreatic cancer indicates that both radiopharmaceuticals exhibit comparable tumor uptake. Following 96 h of *ex vivo* biodistribution with ^177^Lu-TEFAPI-06 and ^177^Lu-TEFAPI-07. ^177^Lu-TEFAPI-07, which incorporates the EB structure, shows significant renal uptake. Finally, intra-tumor radiotherapy with low doses (3.7, 1.85 MBq) was administered into tumors with a volume of 35 mm^3^ for efficacy assessment. The results revealed a pronounced tumor suppression effect for both radiopharmaceuticals. However, the tumor volume in this study was 35 mm^3^, although efficacy was assessed at a low dose, this tumor volume was too small, tumor growth may not have stabilized. Therefore, a conventional volume (50–200 mm^3^) should be used to assess treatment efficacy. This study also illustrates that the introduction of various ABM strategy moieties may result in differences in biodistribution, with IPBA appearing to be more suitable for incorporation into the radiopharmaceutical structure as an ABM strategy.

Meng’s team developed the FSDD_n_I series by utilizing the varying distances between the IPBA and the targeting group (Meng et al., 2022). The ^68^Ga-FSDD_0_I exhibits significant blood circulation retention as observed in micro-PET/CT images. SPECT imaging of ^177^Lu-FSDD_0_I indicates continuous accumulation of radioactivity within the tumor for 96 h. Results from biodistribution experiments demonstrate that intra-tumor uptake peaks at 4 h and tends to decline at 24 h, with a retention rate of 42% at that time. This demonstrates that the installation of an albumin-binding motif and the incorporation of an appropriate steric space allow appropriate pharmacomodulation of radioligands.

Kelly’s team attached p-iodobenzoic acid to the structure of FAPI-02 to bind albumin, utilizing PEG fragments and short peptide fragments as linkers, termed RPS309 (Kelly et al., 2021). *In vitro*, stability experiments suggested multiple peaks of radioactivity in both mouse and human serum for this radiopharmaceutical, which may lead to increased off-target effects of the drug *in vivo*. The SW872 model was selected as the subject for experiments, showing a rapid decrease in *ex vivo* biodistribution within the tumor at 24 h (the retention rate is 34% of that of 4 h). This study did not report head-to-head comparisons with other FAPIs, although it noted a higher intra-tumor uptake of ^177^Lu-RPS at 4 h compared to reported FAPI-04 or FAPI-46. Different tumor models were used in the evaluations, making it challenging to determine the reliability of this comparison.

#### 5.3.2 Evans blue (EB)

Wen’s team introduced truncated EB based on the FAPI-02 structure, designing four compounds, ^177^Lu-EB-FAPI-B1-4, with PEG fragments of varying lengths (Wen et al., 2022). They evaluated ^177^Lu-EB-FAPI-B1 for optimal tumor uptake and biodistribution. Therapy experiment reveals that different doses of ^177^Lu-EB-FAPI-B1 exhibit significant tumor inhibitory effects, highlighting the considerable clinical translational potential of ^177^Lu-EB-FAPI-B1.

#### 5.3.3 Fatty acid

Zhang’s team developed FAPI derivatives that can bind to albumin: FAPI-C12 and FAPI-C16, achieved by linking lauric acid (C12) and palmitic acid (C16) to FAPI-04 (Zhang et al., 2021). Experiments in the HT1080 FAP model showed that the ^86^Y-FAPI-C16 was still well accumulated in the tumor at 48 h. SPECT imaging following ^177^Lu-FAPI-C16 administration revealed significant intra-tumor retention at 72 h (equivalent to 58% at 24 h). The therapeutic efficacy of ^177^Lu-FAPI-C16 was confirmed through treatment experiments in tumor-bearing mice; a higher dose (29.6 MBq) resulted in reduced tumor volume, increased survival rate, and extended survival time.

#### 5.3.4 Covalent strategies

Cui’s team reduced the detachment of the radiopharmaceutical from the target by introducing an irreversible binding structure into the binding site (Cui et al., 2024). Docking simulation studies of the protein were conducted, and analysis of solvents and residues in the FAP binding pocket identified nucleophilic Y450 and neighboring Y210 as potential binding sites for sulfur (VI) fluoride. Several latent covalent including aryl-sulfone fluoride (SF) and aryl fluorosulfate (FS), were selected for incorporation into the structure of FAPI-04. The overly reactive SF resulted in increased radionuclide off-target. FAPI-mFs is considered the best candidate. In patient imaging, the SUV_max_ of ^68^Ga-FAPI-mFs in tumors and the detection rate for some small lymph nodes exceeded that of ^68^Ga-FAPI-04. Tumor treatment experiments at both high and low doses of ^177^Lu-FAPI-mFs demonstrated similar therapeutic effects, indicating that efficacy did not exhibit dose dependence. The efficacy of ^225^Ac-FAPI-mFs in inhibiting tumor growth in the SDC-PDX model was remarkable, with the tumor growth in the high-dose group being completely inhibited, reflecting the significant potential of this radiopharmaceutical as a targeted tumor therapy. This strategy presents a novel approach for developing FAPI derivatives.

#### 5.3.5 Others

Feng’s research team utilized the reaction between maleimide and endogenous albumin’s free thiol groups to carry the radioligand within albumin (Feng et al., 2024). They developed DOTA-FAPI-maleimide by incorporating a maleimide moiety into FAPI-04. The tumor uptake of ^177^Lu-DOTA-FAPI-maleimide in HT1080-FAP models peaked at 24 h post-injection. After 4 days, intra-tumor uptake was 67% of that at 24 h. However, higher uptake was observed in the liver, lung, kidney, and spleen, indicating the need for further optimization of this strategy. Overall, this study developed and evaluated the thiol group-attaching strategy, extending the circulation and tumor retention of the modified FAPI radiopharmaceutical. However, many proteins contain free sulfhydryl groups *in vivo*, which may lead to an increase in non-specific binding sites. Additional data are also needed to support the efficacy/toxicity assessment of this strategy.

Zhou’s research team successfully synthesized three FAPI ligands by optimizing the amino acid linker and incorporating ibuprofen (Zhou et al., 2025). The tumor uptake and retention of ^177^Lu-FAPI-Ibu1, ^177^Lu-FAPI-Ibu2, and ^177^Lu-FAPI-Ibu3 in A549-FAP and U87MG tumors were significantly higher than that of ^177^Lu-FAPI-04. The uptake of ^177^Lu-FAPI-Ibu3 in tumors was 2.5-fold greater than that of ^177^Lu-FAPI-04 in A549-FAP tumor-bearing mice, and the ratios of absorbed dose in tumor-to-liver and tumor-to-kidney were significantly elevated. ^177^Lu-FAPI-Ibu1, ^177^Lu-FAPI-Ibu2, and ^177^Lu-FAPI-Ibu3 significantly inhibited the growth of A549-FAP tumors, with the group treated with ^177^Lu-FAPI-Ibu3 exhibiting the longest survival time, demonstrating a median survival time significantly higher than that of the group treated with ^177^Lu-FAPI-04. These novel radioligands exhibit notable advantages in tumor uptake, retention, and growth inhibition, with ^177^Lu-FAPI-Ibu3 being the most promising candidate.

Hypoxic environments are typically present in tumors compared to normal tissues. Luo’s research team developed this dual-targeted radioligand by incorporating a hypoxia-sensitive nitroimidazole moiety into FAPI-04 (Luo et al., 2024). *In vitro* experiments showed that ^177^Lu-1 exhibited a significantly higher cell binding retention capacity under hypoxic conditions than normoxic conditions, suggesting a “trapping” effect of the nitroimidazole moiety in hypoxic environments. The accumulation of ^177^Lu-1 in the tumor exceeded that of ^177^Lu-FAPI-04. The ^177^Lu-1 exhibited excellent tumor uptake and retention, achieving a high tumor-to-background ratio in both *in vitro* and *in vivo* experiments. These properties resulted in enhanced targeting ability in tumors with elevated levels of hypoxia.

Wang’s team combined FAPI and DOTA using polydopamine (PDA) nanocarriers for specific cancer targeting (Wang et al., 2025). Following local injection at the tumor site, ^177^Lu-DOTA-PEG-PDA-FAPI accumulated significantly, reaching 182.6% ± 73.8% ID/g after 5 days, with stable tumor uptake. Tumor growth was significantly inhibited in the locally injected group, whereas no significant tumor growth inhibition was noted in the intravenously injected group. The median survival time of 36 days in the treatment group was significantly longer than that of the 18 days observed in the control group, indicating a notable therapeutic effect of the radiopharmaceutical. However, intravenous injection was ineffective due to the nanomaterials. The study also identified challenges, such as unstable labeling yield, low specific activity, and a tendency to aggregate during processing, which require further optimization to enhance the clinical application of the radiopharmaceutical. The application of nanomaterials in drug delivery is a rapidly developing field. Its main advantage is its ability to significantly improve drug delivery efficiency, targeting, stability, and bioavailability. Therefore, nanomaterials combined with FAPI are a good design concept. However, the ineffectiveness of conventional drug delivery via intravenous injection in this study presents a major limitation in clinical application. This finding also suggested that the design and selection of nanomaterials is crucial for this strategy.

### 5.4 Multivalent effect

The development of multivalent ligands has been widely designed and applied to enhance the recognition of biomolecules. Multimer optimization strategies have emerged in the development of FAPIs.

Based on the favorable biodistribution and significant tumor uptake of the optimized dimer BiOncoFAP (Galbiati et al., 2022), Galbiati’s team conducted multimer optimization and evaluation, developing the trimeric TriOncoFAP-DOTAGA, tetrameric TetraOncoFAP-DOTAGA, hexameric HexaOncoFAP-DOTAGA, and octameric OctaOncoFAP-DOTAGA (Galbiati et al., 2024). These were evaluated through ^177^Lu labeling, followed by biodistribution studies in SK-RC52.FAP tumor-bearing mice. The trimeric ^177^Lu-TriOncoFAP-DOTAGA and tetrameric ^177^Lu-TetraOncoFAP-DOTAGA demonstrated superior tumor uptake, whereas the tetrameric ^177^Lu-TetraOncoFAP-DOTAGA exhibited improved biodistribution. However, the hexameric ^177^Lu-HexaOncoFAP-DOTAGA and octameric ^177^Lu-OctaOncoFAP-DOTAGA showed significantly higher liver and spleen uptake. The team also incorporated PEG fragments into the TriOncoFAP-DOTAGA structure to increase space, revealing that the longer PEG chains corresponded to lower affinity for FAP. Affinity assessments indicated that TriOncoFAP (IC_50_ = 10.6 nM) exhibited a concomitant increase in affinity for PREP compared to OncoFAP (IC_50_ = 2.9 µM); the affinity of BiOncoFAP for PREP also increased to 302 nM. TriOncoFAP (IC_50_ = 14.8 nM) and BiOncoFAP (IC_50_ = 372 nM) also exhibited higher affinity for DPP 9 compared to OncoFAP (IC_50_ = 6.3 µM), suggesting that this multipotency structural optimization may be accompanied by a decrease in selectivity for FAP.

Zhong’s team utilized lysine as linkers to connect two FAPI-04 groups, forming the dimer DOTA-Suc-Lys-(FAPI-04)_2_ (Zhong et al., 2023). The ^177^Lu-DOTA-Suc-Lys-(FAPI-04)_2_ demonstrated a decreasing trend in tumors over 72 h, with tumor uptake being less than 14% of that at 1 h. However, tumor uptake and retention time were enhanced compared to ^177^Lu-FAPI-04. Multiple treatment courses and two doses were employed to investigate the treatment efficacy. The results indicated that ^177^Lu-DOTA-Suc-Lys-(FAPI-04)_2_ slowed the tumor growth rate, with a higher dose correlating to better efficacy, whereas multiple treatment courses appeared to have mini effect on efficacy.

Hongsheng Li’s team incorporated both DOTA and NOTA into the structure of FAPI-04 dimer to form ND-bisFAPI, aiming for ^18^F/^177^Lu paired labeling, and utilized A549-FAP and U87 models for imaging (Li H. et al., 2022). The biodistribution of ^177^Lu-ND-bisFAPI indicated that tumor uptake increased at 24 h post-injection, followed by a rapid decrease at 72 h. Non-specific uptake in normal organs (joints, lungs, pancreas, liver, spleen, and muscle) was relatively high. Tumor uptake of ^177^Lu-ND-bisFAPI exceeded that of ^177^Lu-FAPI-04. Treatment experiments on A549-FAP models demonstrated significant tumor growth inhibition and prolonged survival, although tumor growth resumed in all experimental mice by the 18th day post-treatment. Tumor growth inhibition was more pronounced with ^177^Lu-NDbisFAPI at the same dose compared to ^177^Lu-FAPI-04, exhibiting a dose-dependent effect.

Building on the effective imaging demonstrated by the previously developed dimer ^68^Ga-DOTA-2P(FAPI)_2_ (Zhao et al., 2022), Zhao’s team hypothesized that increasing the number of target groups might improve ligand binding to the target, thereby extending radiopharmaceutical retention in the tumor (Pang et al., 2023b). They evaluated the tetramer ^68^Ga-DOTA-4P(FAPI)_4_, ^64^Cu-DOTA-4P(FAPI)_4,_ and ^177^Lu-DOTA-4P(FAPI)_4_ for tumor uptake, biodistribution, intra-tumor retention time, and therapeutic efficacy in HT1080-FAP and U87 models. Compared to ^177^Lu-FAPI-46 and ^177^Lu-DOTA-2P(FAPI)_2_, ^177^Lu-DOTA-4P(FAPI)_4_ enhanced tumor uptake and prolonged intra-tumor retention time, allowing U87 tumor-bearing mice to maintain tumor growth for at least 2 weeks without progression.

Millul’s team developed FAPI-46 derivatives, including the monomer FAPI-46-F1, the dimer FAPI-46-F1D, and the two albumin binder derivatives FAPI-46-Ibu with ibuprofen and FAPI-46-EB. These derivatives underwent a head-to-head comparison with FAPI-46 and FAP-2286 to identify the most advantageous strategy (Millul et al., 2023). Following ^177^Lu labeling, SPECT results demonstrated optimal tumor uptake and biodistribution of ^177^Lu-FAPI-46-EB in the high FAP-expressing HT1080.hFAP model, whereas showing poor tumor uptake and biodistribution in the moderately FAP-expressing HEK293.hFAP model. Although ^177^Lu-FAP-2286 did not exhibit the best tumor uptake in HT1080.hFAP tumor models, it demonstrated optimal tumor uptake and biodistribution in HEK293.hFAP tumor models. In the HEK293.hFAP model, ^177^Lu-FAP-2286 achieved the highest AUC at 4 h, while ^177^Lu-FAPI-46-EB maintained the best AUC at 24 h. The study concludes that dimerization of FAP small molecules and cyclic peptides represents two promising strategies to enhance the radiation dose to tumors. The role of albumin-binding agents in regulating distribution *in vivo* is highly dependent on the selection of albumin-binding agents. The tumor uptake and biodistribution of the same radiopharmaceutical can vary significantly across different tumor models. Therefore, validating tumor uptake and biodistribution in multiple tumor models is essential in the development of such radiopharmaceuticals.

The ABM strategies concomitantly increase radiation exposure to normal tissues, most notably the kidneys. Hence, balancing the assessment of RLT efficacy with that of the toxic side effects due to radiation is necessary. The multimerization of the affinity group can increase its ligand to target affinity from nanomolar to picomolar levels, but decrease FAP selectivity due to the concomitant increase in DPPs and PREP affinities, may also lead to increased uptake by some normal organs. Covalent binding strategies are ideal for irreversibly binding radionuclides to target proteins, but these active covalent motifs are not selective and may react with other proteins *in vivo*, resulting in additional radiation exposure to non-target regions. Radioligands for therapeutic applications require higher tumor targeting and affinity requirements than imaging. Therefore, designs accounting for increased uptake in the tumor region and reduced accumulation in healthy tissue are critical for RLT. The development of FAPI molecules for oncology therapeutics still requires further exploration and comparative studies.

Dual-targeted radiopharmaceuticals are also extensively researched for imaging. FAPI-RGD and FAP-RGD, which dimerize with RGD peptide, exhibit high tumor uptake with favorable distribution (Zang et al., 2022; Liu et al., 2024). FAPI-PSMA is used for prostate cancer diagnosis, and showed improves uptake in prostate tumors (Wang et al., 2023; Hu et al., 2022). Furthermore, FAPI-LM3 also showed promising results in the imaging of nasopharyngeal cancer (Zhao et al., 2024a). However, no pertinent treatment evaluation studies have been conducted. Employing these radiopharmaceuticals for the RLT of specific tumors will also furnish new RLT radioligands for these patients.

## 6 Clinical evaluation of targeted FAP radiopharmaceuticals in tumor therapy

Successful preclinical practice of FAPIs positions them as well-suited for clinical targeted radioligand therapy (RLT). Several preclinical studies and clinical case series have reported using targeted FAP peptides and small molecule compounds for the initial exploration of RLT.

### 6.1 FAPI-46 for tumor patient treatment

^90^Y-FAPI-46 was utilized for the evaluation of treatment efficacy and safety in patients with advanced sarcoma, pancreatic cancer, or other cancers (Ferdinandus et al., 2021; Fendler et al., 2022). Approximately 40% of % of the patients achieved stable disease. Some of them experienced blood-related side effects, however, no acute toxicity was observed during treatment. In a clinical trial involving ^177^Lu-FAPI-46 administration to patients with advanced-stage tumors who could not receive other treatments, only one patient developed anemia. The remaining patients tolerated the treatment well. Two patients died due to disease progression during treatment, 60% maintained stable disease, and 30% experienced disease progression (Assadi et al., 2021).

Although these studies demonstrated the potential efficacy of ^90^Y/^177^Lu-FAPI-46 in patients with advanced-stage tumors, a study of on the administration of ^177^Lu-FAPI-46 to a patient with advanced-stage metastatic pancreatic cancer reported a gradual decrease in ^177^Lu-FAPI-46 aggregation in the lesion area over time. A SPECT scan on day 6 indicated the almost complete elution of the radioligand, illustrating that ^177^Lu-FAPI-46 is not the optimal radiopharmaceutical for therapeutic applications (Kaghazchi et al., 2022). This RLT of ^90^Y/^177^Lu-FAPI-46 provided preliminary data, illustrating the potential of FAPI for oncologic RLT and emphasizing the elution of FAPI-46 at the tumor site over time as a crucial factor influencing its efficacy.

### 6.2 FAP-2286 for tumor patient treatment

The study of peptide-targeted radionuclide therapy (PTRT) using ^177^Lu-FAP-2286 evaluated the efficacy of ^177^Lu-FAP-2286 in patients with different tumors (advanced sarcoma (Banihashemian et al., 2024), advanced pancreatic, breast, rectal, or ovarian adenocarcinoma (Baum et al., 2022)), These patients experienced a reduction in primary tumor volume and pain relief after treatment, improving the quality of life for late-stage cancer patients, with acceptable side effects. These preliminary PTRT targeting FAP also offer guidance for future clinical applications and provides additional data for RLT targeting FAP.

### 6.3 Other FAPI derivatives for tumor patient treatment

Several studies have reported on the therapeutic efficacy of novel FAPI radiopharmaceuticals optimized. In a Phase II clinical trial utilizing ^177^Lu-LNC1004 in the treatment of patients with advanced metastatic cancer, 20% of the patients achieved a partial response and 45% in stable disease. 21% of the patients were observed to have hematotoxicity. Improved progression-free survival (PFS) and overall survival (OS) in more than 50% of patients (Fu et al., 2025).

Some studies assessing the comparative efficacy and biodistribution of ^177^Lu-DOTA.SA.FAPi and ^177^Lu-DOTAGA.(SA.FAPi)_2_ (Ballal et al., 2021a; Yadav et al., 2023), patients experienced varying degrees of clinical symptoms and pain relief. In preliminary clinical studies involving patients with medullary thyroid cancer, the dimer ^177^Lu-DOTAGA.Glu.(FAPi)_2_ was compared with the first-generation dimer ^177^Lu-DOTAGA.(SA.FAPi)_2_ regarding tumor and non-target organ uptake. The compound ^177^Lu-DOTAGA.Glu.(FAPi)_2_ exhibited high uptake and prolonged retention time in tumors (Martin et al., 2023). The novel FAPi dimer demonstrated significant advantages in radiolabeling, *in vitro* affinity, and *in vivo* pharmacokinetics, particularly with reduced uptake in normal organs.

Several studies using ^177^Lu-LNC1004 (Fu et al., 2023), ^177^Lu-DOTAGA.(SA.FAPi)_2_ (Ballal et al., 2021b), and ^177^Lu-DOTAGA.FAPi dimers (Ballal et al., 2025) to treat patients with iodine-refractory thyroid cancer have shown partial remissions, pain relief and reduced thyroglobulin (Tg) levels in these patients. Treatment-related adverse events primarily included hematologic toxicities. This may offer a new treatment option for patients with iodine-refractory thyroid cancer who have exhausted all other therapies. However, further validation through prospective clinical trials with larger samples is required to ascertain the precise efficacy of these radiopharmaceuticals.

Although the 90% stromal component of solid tumors makes RLT targeting FAP highly promising, recent studies have indicated that normal organs in mice exhibit a certain level of FAP expression (including pancreas, thyroid, and bone) (Bilinska et al., 2025). In addition, multimerization may reduce the ligand selectivity for FAP (Galbiati et al., 2024), which may account for the physiological increase in the uptake of some nontargeted areas (including the pancreas, salivary glands, and thyroid gland) in clinical patients (Zhao et al., 2022; Zang et al., 2022). Utilizing these radioligands for radiotherapy can increase radiation-induced damage to these organs. In FAPI-related RLT for patients with these advanced-stage tumors, multiple courses of administration were used to maintain the aggregation of radioactivity in the tumor. Notwithstanding the blood-related adverse events in some patients, no acute toxicity was observed during treatment. However, such multiple courses of administration increase the burden on the kidneys and other organs. Further evaluation is required to ascertain the long-term toxicity associated with the treatment.

The clinical evaluation of the effect of FAPI treatment is relatively complex, and uniform and accurate assessment standards and methods are lacking. The size, morphology, metabolism, and other indicators of the tumor may change throughout the treatment. However, the relationship between these changes and the treatment effect remains unclear, bringing a certain degree of difficulty in accurately evaluating the treatment effect. Furthermore, the relevance of changes in certain tumor biomarkers after radiotherapy with FAPI has not been reported. An assessment of tumor progression using biomarkers may provide substantial evidence for efficacy evaluation.

## 7 The evaluation of combined treatment with ^177^Lu-FAPI

The combination of RLT targeting FAP with other anti-tumor therapies may exert a direct and indirect dual effect on malignant cells, thereby significantly enhancing the anti-tumor response. Zhu’s team evaluated the efficacy and feasibility of combining ^177^Lu-DOTAGA.(SA.FAPi)_2_ with the CXCR4 antagonist AMD3100 for the treatment of triple-negative breast cancer (Bao et al., 2024). Zboralski’s team explored the efficacy of ^177^Lu-FAP2286 combined with PD-L1 immunotherapy against fibrosarcoma (Zboralski et al., 2023). Chen’s team analyzed the efficacy of ^177^Lu-LNC1004 (Zhao et al., 2024b) and ^177^Lu-DOTA-2P(FAPI)_2_ (Chen et al., 2025) combined with PD-L1 immunotherapy against colorectal cancer. Preclinical studies revealed that this combination treatment demonstrated a synergistic effect and altered the cellular components of the TME particularly increasing tumor infiltration by immune cells. This finding suggested that the combination therapy enhances anti-tumor immunity by altering the TME. Although combination therapy has shown significant synergistic therapeutic effects and may delay drug resistance, extrapolating this therapy from mice to clinical trials requires a focused assessment of dose and toxicity response. The synergistic effects may increase the risk of toxicity. Patient management is also complicated by combination therapy. Therefore, this treatment modality, therefore, requires extensive data to support its further applications.

## 8 Conclusion and prospect

Currently, FAPI has demonstrated favorable pharmacokinetics *in vivo*, with encouraging results in clinical studies as PET tracers in tumor diagnosis and staging. The clinical efficacy of FAP-targeted RLT depends on FAP expression making FAPI PET an essential screening tool for patient selection prior to FAP targeting RLT (Miao et al., 2022).

The field of FAP-targeted radiopharmaceutical development is also undergoing refinement to enhance the efficacy of FAPI. The majority of these studies utilize over-expression tumor model evaluations, which are not comparable due to technological differences that complicate the determination of the most advantageous structure for the optimization strategy. Therefore, in the development of novel FAPI derivatives, head-to-head comparisons are essential in studies. FAPI derivatives have demonstrated efficacy in terms of tumor targeting and biodistribution. Moreover, the removal of DOTA and other chelating radionuclide moieties as targeting moieties for non-radioactive drug delivery may also be of value in the direction of anti-tumor research.

In addition to ^90^Y and ^177^Lu, which are utilized for RLT, other therapeutic radioisotopes with alpha‐emitters, such as ^225^Ac, ^211^At, ^223^Ra, and ^122^Pb are potentially effective to kill malignant cells. Their highly ionizing can cause clustered DNA double‐strand breaks, and their shorter-range reduces radiation damage to nearby normal tissues. Additional data are need to support the safety of using these therapeutic. Precision radiotherapy by delivering these radionuclides to tumors via FAPI will be attempted in the future.

The use of FAPI targeting for RLT as a potential adjuvant treatment can also been indicated to augment the antitumor efficacy of other medications. Combination therapy utilizing FAP-targeted RLT offers novel strategies to address drug resistance in tumor treatment. Various of factors may influence a tumor’s response to FAP targeting for radiotherapy, including FAP expression in the TME, biological behavior of the tumor, and the interaction between the tumor and surrounding normal tissues. The radiotherapeutic response to cancer can vary considerably among different tumor types. Monotherapy often results in limited efficacy and high rates of tumor recurrence, and combination therapy offers significant advantages. In tumors with increased likelihood of fibrosis-driven tumor recurrence, such as glioblastoma (Watson et al., 2024), bladder cancer (Liang et al., 2023), and breast cancer (Watson et al., 2021), surgery or other treatments can lead to tumor resistance due to fibrosis. In addition, the residual malignant cells harbored in the fibrotic areas increase the possibility of tumor recurrence. Therefore, these treatments combined with ^177^Lu-FAPI therapy may significantly reduce the tumor recurrence rate and thus improve patient survival. However, these hypotheses must be confirmed by a large number of preclinical studies. In addition to chemotherapy and immunotherapy, radiotherapy sensitizers may also enhance the efficacy of FAP targeting for anti-tumor RLT.

With the continuous development and learning of artificial intelligence, PET imaging of homologous molecules can predict the individualized dose of therapeutic radiopharmaceuticals and make RLT safer. Most novel ligands in preclinical models demonstrated ^177^Lu-FAPI elution in tumors over time, significantly affecting the efficacy of FAPI-targeted precision radiotherapy in tumors. For optimal efficacy with RLT, the retention of radiopharmaceuticals in the tumor should be close to the radionuclide half-life to ensure maximum radionuclide-killing efficacy, and simultaneous rapid clearance in nontarget tissues. Therefore, optimizing the FAPI molecular structure, enhancing tumor uptake and metabolic stability, and developing more efficient targeting FAP radiopharmaceuticals are essential for advancing this field.

## Abbreviations

FAP, fibroblast activation protein; FAPI, fibroblast activation protein inhibitor; TME, tumor microenvironment; CAFs, cancer-associated fibroblasts; NFs, normal fibroblasts; α-SMA, α-smooth muscle actin; PDGF-β, platelet-derived growth factor receptors-β; RLT, targeted radioligand therapy; PET/CT, positron emission tomography/computed tomography; SPECT, single photon emission computed tomography; PSMA, prostate specific membrane antigen; DPP IV, dipeptidyl peptidase IV; PREP, prolyl endopeptidase; ABM, albumin-binding motif; Cys, cysteines; PEG, polyethylene glycol; AUC, areas under the curve; IPBA, p-iodophenyl moiety; EB, Evans blue; PDX, patient-derived xenograft; SF, aryl-sulfone fluoride; FS, aryl-fluorosulfate; PDA, polydopamine; Tg, thyroglobulin; PFS, progression-free survival; OS, overall survival; CXCR4, C-X-C chemokine receptor type 4; IC50, half maximal inhibitory concentration; Kd, dissociation constant.
